# Cellular reprogramming in farm animals: an overview of iPSC generation in the mammalian farm animal species

**DOI:** 10.1186/s40104-016-0070-3

**Published:** 2016-02-19

**Authors:** J. Ogorevc, S. Orehek, P. Dovč

**Affiliations:** Department of Animal Science, Biotechnical Faculty, University of Ljubljana, Domžale, Slovenia

**Keywords:** Cellular reprogramming, Farm animals, Induced pluripotent stem cells, Pluripotency

## Abstract

Establishment of embryonic stem cell (ESC) lines has been successful in mouse and human, but not in farm animals. Development of direct reprogramming technology offers an alternative approach for generation of pluripotent stem cells, applicable also in farm animals. Induced pluripotent stem cells (iPSCs) represent practically limitless, ethically acceptable, individuum-specific source of pluripotent cells that can be generated from different types of somatic cells. iPSCs can differentiate to all cell types of an organism’s body and have a tremendous potential for numerous applications in medicine, agriculture, and biotechnology. However, molecular mechanisms behind the reprogramming process remain largely unknown and hamper generation of *bona fide* iPSCs and their use in human clinical practice. Large animal models are essential to expand the knowledge obtained on rodents and facilitate development and validation of transplantation therapies in preclinical studies. Additionally, transgenic animals with special traits could be generated from genetically modified pluripotent cells, using advanced reproduction techniques. Despite their applicative potential, it seems that iPSCs in farm animals haven’t received the deserved attention. The aim of this review was to provide a systematic overview on iPSC generation in the most important mammalian farm animal species (cattle, pig, horse, sheep, goat, and rabbit), compare protein sequence similarity of pluripotency-related transcription factors in different species, and discuss potential uses of farm animal iPSCs. Literature mining revealed 32 studies, describing iPSC generation in pig (13 studies), cattle (5), horse (5), sheep (4), goat (3), and rabbit (2) that are summarized in a concise, tabular format.

## Background

Pluripotent stem cells are unspecialized cells that can evolve to all cell types of an adult organism. Until 2006 they could be isolated only from inner cell mass of early stage embryos. Embryonic stem cell (ESC) lines were first established in mouse [1], and subsequently in human from in vitro-derived embryos [2]. In contrast, derivation of ESCs from embryos of farm animal species was inefficient, probably due to limited knowledge about the biology of the different species ESCs (e.g. timing and isolation of primary cultures, recognition of authentic ESC, and sustaining pluripotency and propagation in culture) [3]. Germline transmission, as the most stringent criteria of pluripotency, has been proved only for murine (mouse and rat) inner cell mas (ICM)-derived ESCs, which are considered true (“naïve”) pluripotent ESCs, whereas most human ICM-derived ESC lines share more common characteristics with mouse epiblast-derived stem cells (mEpiSCs), and are considered “primed” pluripotent stem cells (for a detailed explanation see [4]).

For a long time it was believed that differentiation is a one-way process and that fate of somatic cells is irreversible. The discovery that specialised somatic cells can be reversed back to a non-differentiated state came several decades ago when Gurdon [5] injected frog intestinal epithelium cell nuclei into enucleated frog oocytes and showed that normal feeding tadpoles can be developed from transferred somatic cell nuclei. Proof that reprogramming of mammalian cells is possible was obtained with reproductive cloning of several species (e.g. sheep [6]; cattle [7]; goat [8]; pig [9]) via somatic cell nuclear transfer (SCNT). Interestingly, generation of nuclear transfer ESCs (NT-ESC) in human was not possible for a long time; finally, human somatic cells were successfully reprogrammed by SCNT, first resulting in triploid pluripotent cells [10] and latter in normal (diploid) NT-ESCs [11].

The use of embryos (especially human) for ESC derivation faces ethical concerns and is subjected to rigorous legal restrictions. Reprogramming somatic cells by SCNT is technically challenging, laborious, inefficient (especially in human), and requires the use of oocytes. Reproductive cloning / nuclear transfer experiments showed that oocytes obviously contain factors, which are able to reprogram committed cells. Takahashi and Yamanaka identified these factors and developed a method of direct reprogramming of somatic cells that was simple in principle and circumvented the need for embryo or oocyte manipulations [12]. The so called induced pluripotent stem cells (iPSCs) were generated from mouse embryonic fibroblasts by ectopic expression of only four (Yamanaka set – OSKM: Oct4, Sox2, Klf4, and c-Myc) transduced nuclear transcription factors [12]. Human iPSCs were generated soon after, using a set of slightly different (Thomson set - OSNL: OCT4, SOX2, NANOG, and LIN28) transcription factors [13]. After introduction of the direct reprogramming technology in mouse and human iPSCs were established in various animal species, using the same principle. Different combinations of exogenous reprogramming factors and culture conditions were used, dependent on donor cell type and/or species. Insights from such cell reprogramming experiments could provide missing biological information about pluripotent cells in different species (e.g. optimal conditions for propagation), which might eventually enable derivation and propagation of ICM-derived ESC lines in farm animals.

Transcription factor-directed reprogramming enabled generation of ethically acceptable, individuum-specific, pluripotent stem cells that can be derived from different types of somatic cells, including in species where ESC lines aren’t available. iPSCs closely resemble ESCs in their characteristics and represent practically limitless source of pluripotent stem cells, potentially available for autologous cellular therapies, individuum-specific disease modelling and/or drug screening, and research/testing purposes in medicine and developmental biology. However, there are still many obstacles to overcome. For example, researchers found that numerous subtle but substantial genetic and epigenetic differences exist between iPSCs and ESCs [14], which delay the use of iPSCs for transplantation therapies in human. First, it is necessary to assess survival potential, capability of functional integration into tissues, genetic stability, and absence of tumorigenic potential in reprogrammed cells. Development of animal models, which can be used for preclinical research, is therefore of vast importance [15]. Because of more similar body size, physiology, and characteristics of pluripotent cells to human, development of livestock and non-human primate models seems essential to bridge the gap and enable transfer of iPSCs-based therapy procedures, from rodents (e.g. [16]) to the field of human medicine. For example, preclinical demonstration studies were successfully performed in non-human primates in heart [17] and Parkinson’s disease treatment [18], and in heart disease treatment in post-infarcted swine models [19]. In addition to cell transplantation therapies, interspecies chimeras (farm animals with genetically engineered “organ niches”) could be used in the future to make organs from human pluripotent stem cells, using blastocyst or in utero conceptus complementation in organogenesis-disabled animals, for treating patients that require whole organ replacement [20]. However, interspecies complementation has been shown only between mouse and rat [21, 22], whether chimera generation is possible between more distantly related species, due to interspecies boundaries, remained to be determined. Additionally, existing non-rodent ESCs/iPSCs are mostly considered “primed” state pluripotent stem cells and thus not capable of forming chimeras after blastocyst injection.

**Fig. 1:**
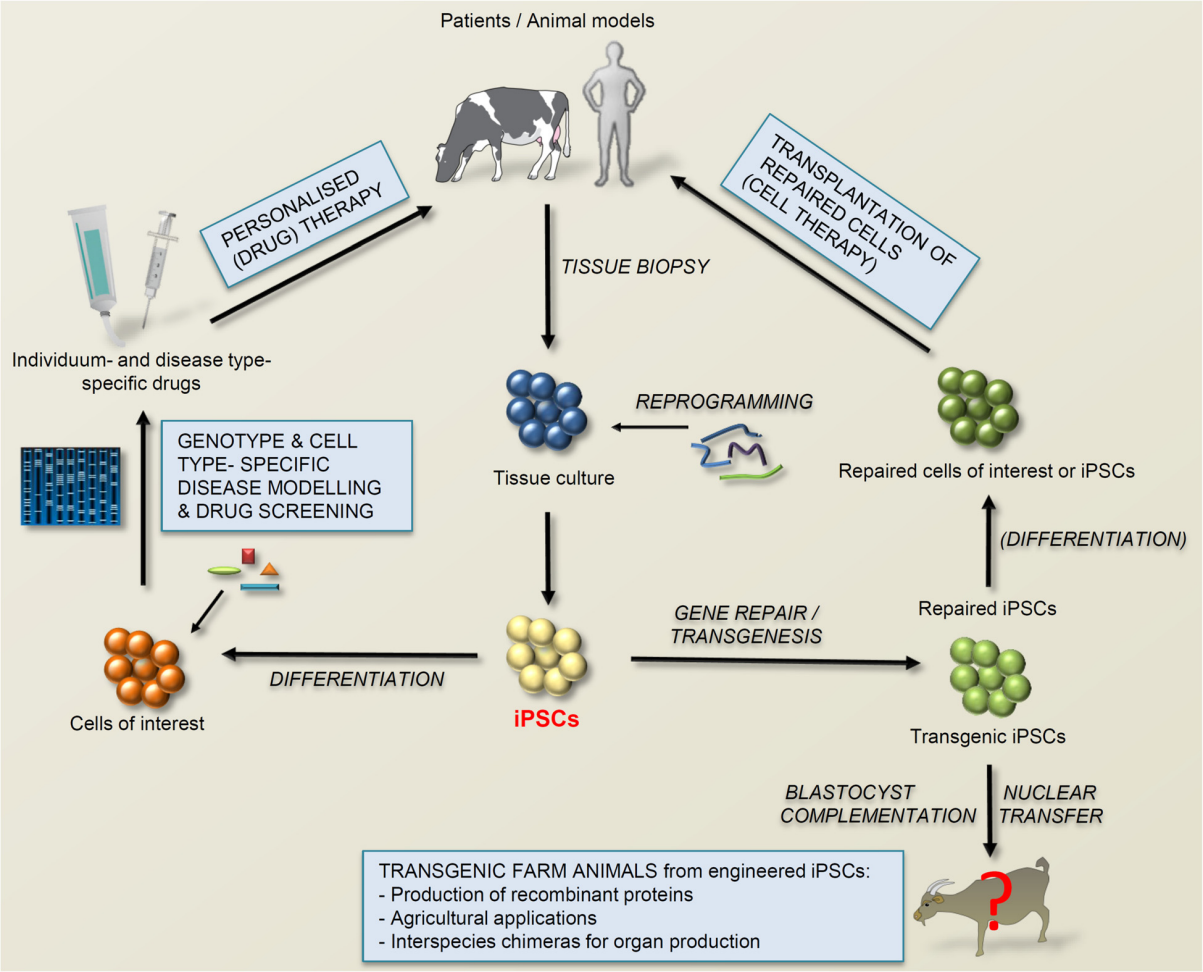
The most promising applicative uses of iPSCs include regenerative cell therapy, personalised disease modelling and drug screening, and generation of transgenic animals from genetically engineered iPSCs that yet needs to be demonstrated in large farm animals. Some of the symbols used in the figure are a courtesy of the Integration and Application Network, University of Maryland Center for Environmental Science (ian.umces.edu/symbols/)

Pluripotent stem cells are not promising only for medical applications, but could found numerous uses in biotechnology and agriculture. Advanced reproduction techniques in farm animals could enable development of genetically modified animals from engineered pluripotent stem cells; SCNT is a method of choice when producing transgenic farm animals [23] and use of genetically engineered pluripotent stem cells (i.e. ESCs or iPSCs capable of generating offspring through nuclear transfer) as donor cells could simplify and improve efficiency of the procedure, as already shown in mice [24]. Transgenic animals could improve agricultural production or be used as bioreactors for production of recombinant proteins. For example, anticoagulant antithrombin, the first marketed recombinant protein produced in transgenic animals [25], is expressed in mammary tissue of genetically engineered goats and isolated from their milk. In agriculture, transgenic animals could improve human health by enhanced nutrition value, help protect the environment, decrease livestock diseases, and increase animal welfare [26]. Potential uses of iPSCs are depicted in Fig. 1.

Reviews on mechanisms and methods of cell reprograming in domestic animals have been published in several articles (e.g. [27, 28]). In the follow up of this article we provide the most recent overview on achievements in iPSC generation in pig, cattle, horse, rabbit, sheep, and goat. Additionally, we provide a comprehensive protein sequence similarity analysis of the most important pluripotency transcription factors (OSKM) between different farm animals, human, and mice, and briefly discuss current trends in reprogramming methodology.

### Comparison of pluripotency-related transcription factors between human, mouse, and farm animal species

Research has shown that human and murine transcription factors can reprogram cells of different mammalian species, as well as of non-mammalian vertebrate and even invertebrate species, pointing to high conservation of pluripotency-related signalling network across a wide phylogenetic range [29]. The cross-species reactivity of pluripotency-related transcription factors, also between distantly related species, indicates fundamentality of the reprogramming principle in biological aspect. To determine conservation of transcription factors across the species of interest we aligned GenBank protein (reference – if available) sequences of the most widely used reprogramming factors (OSKM set) for human, mouse, and the selected farm animal species (cattle, goat, sheep, rabbit, horse, and pig) in ClustalW2 multiple sequence alignment tool (EMBL-EBI: http://www.ebi.ac.uk/Tools/msa/clustalo/). The identity matrices (data not shown) showed that OCT4 protein sequence is the least conserved across the compared species, but still exhibits high sequence similarity; i.e. over 89 % between the farm animals and human and over 84 % when mouse is included to the comparison. SOX2 protein sequence is the most conserved (over 95 % identity in all the compared species), while KLF4 and MYC both exhibited over 90 % identity between all the species. Sequence identity between the large farm animals was around 95 % for all the transcription factors, and reached 98–100 % identity between closely related species (e.g. ruminants).

Human or mouse transcription factors are often used for reprogramming animal cells, either because transcription factor sequences are not available for animal species with un/poorly- annotated genomes and/or because vectors containing human or murine factors are already (commercially) available. Based on the sequence similarities, we suggest that human transcription factors should be used over murine when reprogramming cells of the farm animals (Table 1). However, empirical evidences prove that murine transcription factors can as well be successfully used for reprogramming farm animal cells (e.g. in pig, horse, sheep – data available in Table 2).

**Table 1:** Similarity (%) of OSKM protein sequences of different species to human and mouse (human/mouse)

Species	OCT4	SOX2	KLF4	c-MYC
Pig	93 / 86 [NP_001106531]	99 / 97 [NP_001116669]	96 / 92 [NP_001026952]	93 / 91 [NP_001005154]
Horse	94^a^ / 86^a^ [XP_001490158]	98^a^ / 96^a^ [ACJ12602]	94^a^ / 91^a^ [XP_005605741]	92^a^ / 91^a^ [XP_001498041]
Rabbit	90 / 85 [NP_001093427]	98^a^ / 99^a^ [AJC97786]	96^a^ / 92^a^ [AJC97787]	94^a^ / 92^a^ [XP_008254124]
Sheep	91^a^ / 84^a^ [XP_004019017]	100^a^ / 98^a^ [CAA65725]	96 / 93 [NP_001157691]	93 / 92 [NP_001009426]
Cattle	91 / 84 [NP_777005]	100 / 98 [NP_001098933]	96 / 93 [NP_001098855]	92 / 91 [NP_001039539]
Goat	91 / 84 [NP_001272498]	97 / 96 [NP_001272601]	96^a^ / 92^a^ [XP_005684447]	92^a^ / 92^a^ [XP_005689000]
Human	100 / 86 [NP_002692]	100 / 98 [NP_003097]	100 / 92 [NP_004226]	90 / 100 [NP_002458]
Mouse	86 / 100 [NP_038661]	98 / 100 [NP_035573]	92 / 100 [NP_034767]	100 / 90 [NP_034979]

The GenBank accession numbers are available in square brackets

^a^similarity is based on predicted or non-curated sequences

**Table 2:** Summary of studies describing iPSC generation in the selected farm animal species

Species	Donor cell type	Insertion method	Transcription factors^a^	Culture conditions (growth surface; serum or serum replacements, factors/inhibitors)^b^	Reference
Pig	embryonic fibroblast	retroviral transduction	hOSKM and mOSKM	iMEFs; defined FBS + bFGF	[51]
	fetal fibroblasts	lentiviral transduction	hOSKM	iMEFs; KOSR + bFGF	[52]
	ear fibroblasts and bone marrow cells	Tet-on-inducible lentiviral transduction	hOSKMNL	iMEFs; KOSR	[53]
	mesenchymal stem cells	lentiviral transduction	hOSKMNL	iMEFs; KOSR + bFGF	[45]
	ear fibroblasts	retroviral transduction	hOSKM	iMEFs or gelatin; FBS and KOSR (1:1) + bFGF + LIF	[54]
	ear fibroblasts	retroviral transduction	mSKM	iMEFs or gelatin; FBS and KOSR (1:1) + bFGF + LIF	[55]
	embryonic fibroblasts	Tet-on-inducible lentiviral transduction	hOSKMN	iMEFs; KOSR + bFGF	[56]
	fetal fibroblasts	lentiviral transduction	hOSKM	iMEFs; KOSR + bFGF	[57]
	fetal fibroblasts	sleeping beauty transposon system	mOSKM	iMEFs or iSNLs or gelatin; KOSR + bFGF	[58]
	embryonic fibroblasts	retroviral transduction	hOSKM	iMEFs; FBS and KOSR (1:1) + bFGF + LIF	[59]
	embryonic fibroblasts	retroviral transduction	mOSKM	iMEFs; KOSR + bFGF or FBS + bFGF + LIF or FBS + bFGF + LIF + VPA	[60]
	mesenchymal stem cells	retroviral transduction	pOK + small molecules	iMEFs; KOSR or FBS + LIF	[61]
	fetal fibroblasts	episomal plasmid	hOSKMNL	iMEFs; N-2 suppl. + B-27 suppl. + LIF + GSKi + MEKi	[44]
Horse	fetal fibroblasts	piggyBac transposon system	mOSKM	50 % iMEFs and 50 % iEFFs; FBS + bFGF + LIF + GSKi + TGFi + TZV + ALKi	[49]
	fibroblasts	retroviral transduction	hOSK	iMEFs; FBS + ITS + EGF + bFGF + LIF	[62]
	skin fibroblasts	retroviral transduction	mOSKM	iSNLs; FBS or KOSR + bFGF and/or LIF	[63]
	keratinocytes	retroviral transduction	mOSKM	iSNLs; FBS + bFGF + LIF	[64]
	skin fibroblasts	lentiviral transduction	hOSKM	- reprogramming: Matrigel; ES-FCS + bFGF + LIF + GSKi + MEKi + TGFi + ALKi	[65]
				- putative iPSCs: iMEFs; ES-FCS + LIF or ES-FCS + LIF + bFGF or ES-FCS + LIF + bFGF + MEKi or ES-FCS + LIF + bFGF + PI3K/AKTi or ES-FCS + LIF + bFGF + MEKi + PI3K/AKTi	
Rabbit	liver and stomach cells	lentiviral transduction	hOSKM	iMEFs; KOSR + bFGF + LIF	[66]
	ear fibroblasts	retroviral transduction	hOSKM	iMEFs; KOSR + bFGF	[67]
Sheep	embryonic fibroblasts	retroviral transduction	hOSKM	iMEFs; FBS + ITS + bFGF + LIF	[68]
	ear fibroblasts	Tet-on-inducible lentiviral transduction	hOSKMNL + SV40 T + hTERT	iMEFs; KOSR	[69]
	fetal fibroblasts	Tet-on-inducible lentiviral transduction	mOSKM	iMEFs; KOSR or FBS + bFGF	[70]
	embryonic fibroblasts	retroviral transduction	mOSKM	iSNLs; KOSR + bFGF	[71]
Cattle	fetal fibroblasts	retroviral transduction	bOSKMNL	iMEFs; KOSR + bFGF	[72]
	fetal fibroblasts	lentiviral transduction	hOpSMK	iMEFs; FBS + bFGF + LIF	[73]
	embryonic fibroblasts	poly-promoter plasmid	bOSKM	iMEFs; LIF + MEKi + GSKi	[74]
	ear fibroblasts	retroviral transduction	hOSKMN	iMEFs; FBS + ITS + bFGF + LIF	[75]
	fetal fibroblasts	piggyBac transposon systems	hOSKMNL	iMEFs; KOSR + bFGF + LIF	[76]
Goat	ear fibroblasts	Tet-on-inducible lentiviral transduction	hOSKMNL + SV40 T + hTERT	ND	[77]
	fetal ear fibroblasts	lentiviral transduction	hOSKM	iMEFs; KOSR + bFGF	[78]
	fetal fibroblasts	lentiviral transduction	bOSKMNL + miR- 302/367	- iMEFs; KOSR + LIF + MEKi + GSKi + VPA or KOSR + bFGF + VPA- iSNLs; KOSR + bFGF + VPA	[79]

^a^O OCT4, S SOX2, K KLF4, M MYC, L LIN28, N NANOG, SV40 T Simian vacuolating virus 40 large T antigen, TERT telomerase reverse transcriptase, miR-302/367 microRNA cluster 302/367, h human, m mouse, p pig, b bovine

^b^for a detailed medium composition and culture conditions see reference – only growth surface/feeder layer and the selected growth medium supplements (serum/serum replacements, growth factors, and signalling pathway inhibitors) are presented in the table. *iMEFs* inactivated (mitomycin C) or irradiated mouse embryonic fibroblasts, *FBS* fetal bovine serum, *bFGF* basic fibroblast growth factor, *KOSR* knockout serum replacement, *LIF* leukemia inhibitory factor, *iSNLs* inactivated transformed mouse fibroblasts with expression of leukemia inhibitory factor, *VPA* valproic acid, *iEFFs* inactivated equine fetal fibroblasts, *GSKi* glycogen synthase kinase inhibitor, *MEKi* mitogen-activated protein kinase kinase 1 inhibitor, *TGFi* transforming growth factor-beta inhibitor, *TZV* thiazovivin, *ALKi* anaplastic lymphoma kinase inhibitor, *ITS* insulin/transferrin/selenium supplement, *EGF* epidermal growth factor, *ES-FCS* embryonic stem cells-qualified fetal calf serum, *PI3K/AKTi* phosphatidylinositol 3-kinase/protein B kinase (AKT) inhibitor, *ND* no data

### Current trends in reprogamming methodology

iPSCs were first generated using viral-based (mostly lentiviruses and retroviruses) transduction of transcription factors into the genome of donor cells. The use of genome-integrating methods using viral transduction remains a gold standard in iPSC generation. However, new methods, which surmount genome interventions (so-called “non-integrating techniques”) are being extensively developed and evaluated. Several articles describing reprogramming methods were published in the last years and we recommend them for further reading to readers interested in a more detailed review of the reprogramming methodology (e.g. [30, 31]).

Current trends in reprogramming technology are directed to integration- and feeder-free procedures; the former preventing occurrence of insertional mutagenesis and the latter contamination of donor cells with murine feeders (xeno-free conditions). Inactivated mouse embryonic fibroblast feeders are being replaced by defined components of extracellular matrix, circumventing the need for co-culture. Non-integrating vectors (e.g. adenoviruses, episomes), integrating vectors exhibiting subsequent excision (e.g. piggyBac transposons), or direct input of small molecules, mRNA or proteins into donor cells are being used to bypass genome insertions. Alternatively, studies report that (partial) dedifferentiation of mammalian somatic cells is also possible by cell fusion [32] or addition of extracts to the medium [33–37], however these methods are highly inefficient and usually don’t result in stable iPSC lines.

Recent reprogramming methods are focusing on improving mRNA-based procedures [38], for example by employing synthetic self-replicative RNA that circumvents the need for repetitive transfections [39]. With growing knowledge on reprogramming the reduction in number of ectopically expressed transcription factors was achieved that largely depends on donor cell type. In some cases cells were successfully reprogrammed using ectopic expression of only one transcription factor – for example, only OCT4 “master gene” was sufficient to reprogram neural stem cells [40]. Furthermore, it was shown that cells could be reprogrammed without exogenous transcription factors delivery, by using certain chemical compounds that can substitute for transcription factors. At first, such compounds were used in combination with ectopic expression of transcription factors in order to improve reprogramming efficiency or substitute for some of the transcription factors, but usually at least expression of exogenous OCT4 was required (e.g. [41, 42]). However, in 2013 a group of Deng and colleagues succeeded to reprogram mouse somatic cells, by using only a cocktail of seven chemical compounds and called the reprogrammed cells CiPSC – chemically induced pluripotent stem cells [43].

### iPSCs in the farm animals

Literature mining revealed 32 studies describing iPSC generation in the farm animals included in the search (13 in pig, 5 in horse, 5 in cattle, 4 in sheep, 3 in goat, and 2 in rabbit) (Table 2). Different insertion methods and sets of transcription factors were employed in iPSC generation of the selected farm animal species. Table 2 represent a concise overview of publications (until 9/2015) regarding iPSC generation in mammalian farm animal species (cattle, pig, goat, sheep, horse, and rabbit).

The studies show that species-specific, human, mouse or combinations of transcription factors from different species can be used for reprogramming farm animal cells. In most cases OSKM set was sufficient to reprogram donor cells of interest. In several cases NANOG and LIN28 factors were added to the reprogramming cocktail and/or expression of nuclear transcription factors was combined with either addition of small chemical compounds or expression of Simian virus 40 large T antigen (SV40 T), catalytic subunit of human telomerase reverse transcriptase (hTERT), or micro RNA cluster 302/367 (miR-302/367) to achieve higher reprogramming efficiency and stability of iPSC lines. It seems that especially cells originating from ruminant species require expression of additional factors – e.g. NANOG, LIN28, and/or SV40 T and hTERT. Additionally, growth surface and reprogramming medium with its supplements (e.g. growth factors, inhibitors) play an important role in reprogramming efficiency.

Mostly integrating (viral transduction- or piggyback transposons-based) methods were used for reprogramming farm animal cells, except for episome-based, non-integrating method, used for reprogramming pig fibroblasts [44]. There are no reports of the more up-to-date methods (e.g. non-integrating virus- or mRNA-based) being used for reprogramming cells in farm animal species. The expression of the delivered exogenous factors was in most cases controlled by a constitutive promoter (e.g. CMV, EF1α) or in some cases by inducible tetracycline controlled transcriptional activation. Fibroblasts were commonly used as a starting cell type, probably because they are easily accessible and maintained in the culture, and previously validated in reprogramming experiments in human and mouse. In the future, different somatic cell types should be used to reveal, which cell type is optimal for production of *bona fide* iPSCs.

Pluripotent character of the reprogrammed cells in the collected publications was in most cases assessed by determining expression of specific markers, *in vitro* differentiation, and teratoma formation in immunodeficient mice. Germline contribution potential was not tested or the tested cells were not germline competent, therefore the majority of the iPSCs do not meet pluripotency criteria in the most stringent sense and should be considered iPSC-like cells; except for a pig iPSC line [45], where chimeric pigs, demonstrating germline transmission were reported to be produced from the iPSCs [46]. However, proof was based only on a PCR test that showed high blastocyst complementation (indicating successful incorporation of iPSCs), but germline transmission of chimeric pigs to progeny was very low (only two piglets out of 43 were transgenic). At the moment there are no large animal iPSCs that could reliably produce viable and fertile offspring, possibly because of inability to produce stable transgene-free iPSCs, without sustained expression of exogeneous transcription factors [44]. The reliable assessment of pluripotency is one of the main issues in the field, especially when dealing with human iPSCs, where *in vivo* tests cannot be performed for obvious ethical reasons. The situation calls for revaluation and standardisation of minimal pluripotency criteria in different species, which should be indisputably proven, prior a study claiming generation of iPSCs could be published.

Regarding number of publications pig has been the most intensively studied farm animal. Pig with its organ size and physiology represents the best available approximation to human [47] and is a valuable model for testing new therapeutic approaches before they can be introduced into clinics. Stem cell research has been extensively focusing on rodent models, which didn’t prove optimal for testing human therapeutic applications, therefore utilization of large animal models has a great potential to expand our knowledge and is expected to increase [48]. For example, equine iPSCs could be used as a model for pre-clinical validation of stem cell therapies for muscles, joints, tendons, ligaments, and bone injuries, which were extensively studied and treated in sport horses, using mesenchymal stem cells [49].

Transgenic mammals (especially those used for milk production), generated by blastocyst complementation or nuclear transfer from genetically modified stem cells, could be used for large scale production of recombinant proteins of biomedical/biotechnological interest. Expression of transgenic proteins in mammary gland is currently the most optimal production systems, because it allows recombinant proteins to be relatively easy isolated from milk [50]. On the other hand, developed transgenic technology, small size, and short gestation period makes rabbits a lower-cost alternative to ruminants in transgenic milk expression systems, especially suitable for molecular pharming on a smaller scale [50].

## Conclusions

This review focuses on iPSC generation in farm animals and summarizes the research in the field done so far. Based on the literature review we conclude that although their indisputable potential in biotechnology and agriculture or as models for preclinical research iPSCs in farm animals haven’t received the deserved attention. For example, there are thousands of studies focusing on cell reprogramming in human and murine, but we found only 32 studies describing cell reprogramming in the most important mammalian farm animal species. The promise of cell therapies in human medicine seems by far the most appealing application of iPSCs. However, many obstacles will have to be overcome before iPSC-based treatments could be introduced into the clinical practice. Farm animals represent a valuable model for development and testing of such transplantation procedures. Additional attention should be directed to other uses of iPSCs in farm animals – e.g. biopharming and agricultural applications that seem to be (unjustifiably) outshined by the potential of regenerative medicine applications. With this review we wanted to summarise the achievements of cell reprogramming in the farm animals and encourage further studies in this promising field of science.
